# Assessing Cardiovascular Risk with Coronary Artery Calcium and Carotid Intima-Media Thickness in Patients with Negative Stress Echocardiography

**DOI:** 10.3390/biomedicines12092151

**Published:** 2024-09-23

**Authors:** Narae Kim, Hui-Jeong Hwang, In-Ho Yang

**Affiliations:** Department of Cardiology, Kyung Hee University College of Medicine, Kyung Hee University Hospital at Gangdong, Seoul 05278, Republic of Korea; knr802@naver.com (N.K.);

**Keywords:** treadmill echocardiography, coronary artery calcium score, carotid intima-media thickness, cardiovascular outcomes

## Abstract

**Background**: The role of treadmill stress echocardiography (TSE) in symptomatic patients may be limited. We evaluated whether carotid intima-media thickness (cIMT) and coronary artery calcium (CAC) scores can predict cardiovascular (CV) outcomes in patients with negative TSE. **Methods**: Patients who had negative TSE and measured cIMT or CAC scoring were enrolled and followed up. The primary CV outcome was defined as a composite of acute coronary syndrome, coronary revascularization, heart failure, stroke, and CV death. **Results**: Overall, 1095 patients participated. The median follow-up duration was 5.8 years. Patients with increased cIMT and CAC scores experienced a high incidence of primary CV outcomes (normal vs. increased group on cIMT and CAC scoring: 4.4% vs. 20.0% and 0.4% vs. 25.0%, respectively, *p* < 0.001). In the Cox proportional hazard model, increased cIMT and CAC scores were associated with increased primary CV outcomes (adjusted hazard ratio [95% confidence interval], *p*-value for increased cIMT and increased CAC scores = 2.939 [1.241–6.960], *p* = 0.014 and 45.192 [5.497–371.505], *p* < 0.001, respectively). **Conclusions**: Patients with increased cIMT and CAC scores have poor CV outcomes even though they have negative TSE results, and therefore, they should be carefully monitored.

## 1. Introduction

According to the World Health Organization, cardiovascular disease (CVD) stands as the most common cause of mortality worldwide, claiming more than 18 million lives annually, which accounts for one-third of all global deaths [1]. CVD is closely related to the progression of atherosclerosis. Thus, early detection of significant atherosclerosis before it progresses to CVD is important. However, current treatment guidelines consider factors such as age, sex, race, smoking, and underlying diseases for CVD risk estimation without factoring in the presence or absence of atherosclerosis in imaging [2].

Treadmill stress echocardiography (TSE) is a useful test for diagnosing coronary artery disease (CAD) [3]; the prognosis for patients with negative TSE is usually considered to be good [4]. Nonetheless, TSE has inherent limitations as a functional study, particularly that it cannot differentiate subclinical atherosclerotic diseases from normal coronary arteries [5]. Furthermore, acute coronary syndrome does not necessarily result from significant chronic luminal narrowing; however, it may be provoked by spasm, plaque rupture, and thrombus [6].

Carotid intima-media thickness (cIMT) is a marker for the early stages of atherosclerosis [7]. cIMT can predict CV risk, and some studies suggest increased cIMT is associated with CVD in patients with inconclusive treadmill test results [7,8,9,10,11]. However, unlike carotid plaques, which have independent predictive value for CV risk, the relative importance of cIMT on CV risk is still controversial [7]. In several studies, adding cIMT did not improve CV risk stratification [7,12]. In addition, data on patients with symptomatic negative TSE are scarce.

Carotid intima-media thickness (cIMT) is a marker for the early stages of atherosclerosis [7]. Some studies suggest increased cIMT is associated with CVD in patients with inconclusive treadmill test results [7,8,9,10,11]. However, unlike carotid plaques, which have independent predictive value for CV risk, the relative importance of cIMT on CV risk is still controversial [7]. In several studies, adding cIMT did not improve CV risk stratification [7,12]. In addition, data on patients with symptomatic negative TSE are scarce.

Coronary artery calcium (CAC) scoring is a test to quantify morphological abnormalities of atherosclerosis [5,9]. An increased CAC score is related to CV risk, and recent guidelines on dyslipidemia recommend that a CAC score can be considered for CV risk assessment and treatment [2,13]. In the 2019 European Society of Cardiology/European Atherosclerosis Society (ESC/EAS) guidelines for the management of dyslipidemias, the CV risk increases when the CAC score is ≥100 [2]. The 2018 American Heart Association/American College of Cardiology (AHA/ACC) guideline on the management of blood cholesterol recommends the use of statins in patients with a CAC score of ≥100, or a score of 1–99 over 55 years of age [13]. In addition, some studies combined the treadmill test and CAC score to predict CV risk, and previous studies showed that the CAC score had an additional predictive role in patients with positive TSE [14,15]. However, their clinical implications have not been established in symptomatic patients with negative TSE.

Among studies on the relationship between cIMT or CAC score and CV outcome, data combined with the treadmill test for symptomatic patients are insufficient. In this study, we conducted a study on symptomatic patients with a negative TSE. We aimed to evaluate whether increased cIMT and CAC scores can predict CV outcomes in patients with chest pain but with negative TSE.

## 2. Materials and Methods

### 2.1. Patient Population

**Figure 1:**
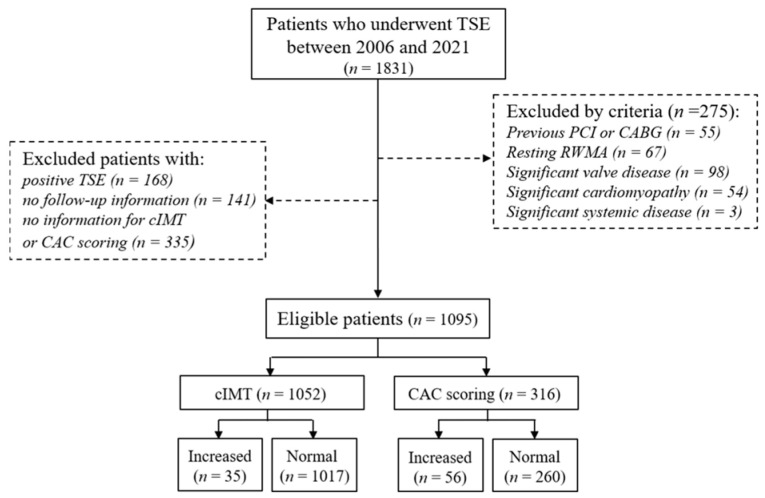
Study population. TSE, treadmill stress echocardiography; cIMT, carotid intima-media thickness; CAC, coronary artery calcium; PCI, percutaneous coronary intervention; CABG, coronary artery bypass graft; RWMA, regional wall motion abnormality.

We recruited patients who had angina symptoms and underwent TSE between November 2006 and August 2021 at our hospital (Kyung Hee University Hospital at Gangdong, Seoul, Republic of Korea). Among them, patients who underwent cIMT or CAC scoring tests within 7 days were enrolled (Figure 1). Patients with positive TSE findings were excluded. Patients aged < 20 years with regional wall motion abnormalities, previous history of percutaneous coronary intervention or coronary artery bypass grafting, valvular heart diseases of greater than moderate grade, significant cardiomyopathy such as heart failure, and severe systemic diseases such as chronic obstructive pulmonary disease, hepatic failure, and end-stage renal disease were also excluded. Informed consent was acquired verbally during the telephone interview. This study received the approval of the hospital Ethics Committee (KHNMC 202211011).

### 2.2. Treadmill Stress Echocardiography

TSE was performed using the standard Bruce protocol with symptom-limited exercise interruption. Tests were performed while monitoring 12-lead electrocardiography, blood pressure, and heart rate. Baseline echocardiography was taken at the left decubitus position before exercise and left ventricular wall motion was compared with postexercise echocardiography at the left decubitus position. A normal unchanged echocardiographic result signified a negative TSE. Angina was defined as chest pain or dyspnea developed during exercise. 

### 2.3. Carotid Artery Ultrasound

A carotid artery ultrasound was concomitantly performed during echocardiography before exercise. The cIMT was measured at both common carotid arteries in the supine position using a high-resolution B-mode ultrasound system (iE33 Ultrasound with Linear Array Transducer, Philips, San Jose, CA, USA) according to a standardized protocol; it was obtained in the posterior wall of the common carotid artery 1 cm below the carotid artery bifurcation in the longitudinal view. The mean cIMT was calculated as a mean value of bilateral cIMT and was defined to be increased when the mean cIMT was >0.90 mm. All measurements were performed by experienced operators blinded to the patient’s information.

### 2.4. Coronary Artery Calcium Scoring

The CAC scores were measured with a 64-slice multi-detector computed tomography (CT) system (Brilliance 64 CT, Philips Medical System, Cleveland, OH, USA). CT was performed with a total voltage of 120 kV, an effective current of 800 mAs, a gentle rotation time of 200 ms, and a 64 × 0.625 mm slice configuration setting. The CAC score was measured through Agatston scoring, which was defined to be increased when the CAC score was measured above 75. A detailed rationale will be provided in the discussion.

### 2.5. Follow-Up and Cardiovascular Outcomes

Patients were divided into both groups according to cIMT and CAC scoring: (1) increased (>0.9 mm) vs. normal (≤0.9 mm) groups on cIMT; (2) increased (>75) vs. normal (≤75) groups on CAC scores. Acute coronary syndrome, coronary revascularization, stroke, heart failure, and CV death were evaluated as CV outcomes. The primary outcome was the composite of CV outcomes. Clinical characteristics, TSE results, and follow-up data for CV outcomes were acquired through medical records and telephone interviews.

### 2.6. Statistical Analysis

Statistical analysis was performed with SPSS version 13.0. Continuous variables were expressed as mean ± standard deviation (or median value [25th percentile; 75th percentile] for variables with skewed data). The categorical variable was demonstrated as a percentage of the group. Student’s *t*-test (or the Mann–Whitney test for variables with skewed data) was used to compare continuous variables in the two groups. χ^2^-test (or Fisher’s exact test for cell counts < 5) was used to compare categorical variables. The cutoff value of the CAC score was determined as a value with high sensitivity and specificity using the receiver operating characteristic (ROC) curve. Incidence rates for the primary outcomes in increased cIMT and CAC groups were compared with those in normal groups using Kaplan–Meier curves and log-rank tests. Univariable and multivariable Cox proportional hazard models were used to evaluate whether increased cIMT and CAC scores are predictors of CV outcome. Values of *p* < 0.05 were considered statistically significant.

## 3. Results

A total of 1095 patients with negative TSE were eligible and enrolled (Figure 1). Among them, patients who underwent cIMT and CAC scoring tests were 1052 and 316, respectively. The median follow-up time was 5.8 (2.4; 8.3) years. Patients with increased cIMT were older and had higher body mass index (BMI), hypertension, diabetes, and dyslipidemia and took more antiplatelet agents, angiotensin-converting enzyme inhibitor (ACEI)/angiotensin receptor blocker (ARB), and statins (Table 1). Patients with increased CAC scores were older, had a higher incidence of hypertension and dyslipidemia, and took more antiplatelet agents, ACEI/ARBs, and statins (Table 1). The mean cIMT was 0.62 ± 0.11 mm in the normal group and 0.98 ± 0.07 mm in the increased group; the CAC score was 0 (0; 5.2) in the normal group and 180.8 (118.15; 410.1) in the increased group (Table 2). The primary outcome was higher in increased cIMT (4.4% in the normal group vs. 20.0% in the increased group, *p* < 0.001) and CAC (0.4% in the normal group vs. 25.0% in the increased group, *p* < 0.001) groups (Table 2).

**Figure 2:**
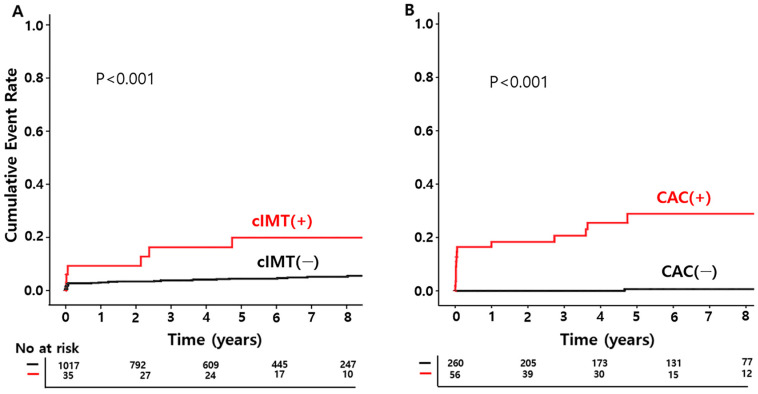
The cumulative incidence of the primary outcomes: A. cIMT, B, CAC scoring. cIMT, carotid intima-media thickness; CAC, coronary artery calcium score; (−), normal group; (+), increased group.

On comparing the cumulative incidence of the primary outcome in the cIMT-increased group and the cIMT-normal group using a Kaplan–Meier plot, significantly more CV outcomes occurred in the cIMT-increased group than in the cIMT-normal group (*p* < 0.001, Figure 2). Similarly, the occurrence of the primary outcome was higher in the CAC-increased group (*p* < 0.001, Figure 2).

Most CV outcomes occurred within 3 months of examination, and except for one stroke, all early events were due to coronary revascularization. Most cases of coronary revascularization (39/50) had been carried out within 1 year after the TSE test. The coronary angiographic findings of these patients showed most patients had intermediate lesions (50–90%) in the mid or distal coronary artery or lesions in branch arteries such as the obtuse marginal artery (OM) (Table S1).

In univariable analysis of the Cox proportional hazard model, the primary outcomes were high in older persons, male, patients with hypertension and diabetes, high systolic and diastolic blood pressures, low heart rate and target heart rate, and patients with angina during the test, and increased cIMT and CAC score (Table 3, Table S2). In multivariable analysis of the Cox proportional hazard model, age, sex, history of hypertension and diabetes, blood pressure, heart rate, target heart rate, and angina during the TSE test were adjusted. Increased cIMT and CAC scores were independent predictors of the occurrence of the primary outcome (hazard ratio [HR] = 2.939, 95% confidence interval [95%CI] = 1.241–6.960, *p* = 0.014 in the cIMT group; HR = 45.192, 95% CI = 5.497–371.505, *p* < 0.001 in the CAC group).

## 4. Discussion

We evaluated the relevance of increased cIMT and CAC scores in predicting CV outcomes among patients presenting with angina symptoms but with negative TSE tests. Our findings suggest that increased cIMT and CAC scores may serve as useful markers for predicting CV outcomes even in symptomatic patients with negative TSE results. The key findings from this study are as follows: First, compared to the normal group, patients with increased cIMT or CAC scores are older, have a higher BMI, and have underlying diseases. Second, patients with increased cIMT or CAC scores exhibit a significantly increased incidence of CV outcome. Third, in patients with angina with negative TSE test results, increased cIMT and CAC scores are independent risk factors for the occurrence of CV outcomes.

Patients with increased cIMT or CAC scores can be considered to have atherosclerosis. Age, obesity, and underlying CV diseases are well-known risk factors for atherosclerosis. This study also demonstrated that the group with an increased cIMT or CAC score was older, had a higher BMI, and possessed underlying CV diseases compared to the normal group. Patients with increased cIMT or CAC scores took more antiplatelets, ACEi/ARB, and statins. This is because patients with atherosclerosis had underlying CV diseases rather than the progression of atherosclerosis occurring despite taking medications. On the other hand, some diseases are strongly related to CV risk. Myeloproliferative neoplasms are diseases associated with high CV risk by increasing thrombotic risk and worsening CV complications due to genetic mutations and chronic inflammation [16]. Rheumatic diseases such as systemic lupus erythematosus or rheumatoid arthritis are well known to increase CV risk by inducing a prothrombotic state due to an inflammatory response. [17,18]. This study excluded patients with such severe systemic disease to prevent bias.

The cutoff value of cIMT and CAC score in this study was determined based on previous data. Setting a cutoff value for cIMT is very controversial, and the normal range for cIMT varies with age [7]. However, according to the 2023 ESH guidelines for the management of arterial hypertension, cIMT above 0.90 mm is considered abnormal [7]. Based on the guidelines, when the mean cIMT exceeded 0.90 mm, it was classified as increased in this study. As for the CAC score, recent guidelines have indicated that CV risk is elevated regardless of other factors when the CAC score is ≥100 [2,13]. Previous studies have often set the cutoff value of the CAC score at 100 [19]. However, existing studies have suggested that a CAC score of zero holds significance in symptomatic patients [20]. Considering these studies, we set the cutoff value of the CAC score to 75 through the ROC curve analysis (Figure S1).

Despite the varied data regarding the diagnostic accuracy of TSE, the recent EVAREST observational study [21] reported a high accuracy rate of 95%. This suggests that patients with negative TSE results are likely to be without CAD. However, issues with the diagnostic accuracy of TSE may have affected the results because most CV outcomes in this study occurred within a short period after the TSE test. The primary reasons for early coronary angiography after the TSE test were abnormal findings on coronary CT angiography or myocardial single photon emission CT. Most coronary angiographic findings for early events showed intermediate lesions in the distal portion of the coronary artery or lesions in branch arteries (Table S1). The ischemic burden due to these lesions was not significant, and it is presumed that these patients had negative results in the TSE test. Therefore, the increase in CV outcomes in patients with increased atherosclerotic markers suggests that increased cIMT and CAC scores may help predict CVD in symptomatic patients even if TSE is negative.

In this study, the increased cIMT was identified as an independent predictor of CV outcomes. Some studies have suggested that cIMT does not have an independent effect on CV risk, and current guidelines do not recommend routine use of carotid imaging [7,12,13]. However, a recent meta-analysis suggested that reducing the progression of cIMT using antihypertensive, lipid-lowering, and antidiabetic drugs reduced the risk of CV outcome [22]. The mean value of cIMT measured in the common carotid artery was related to CV risk, and medication for the progression of cIMT was associated with reduced CV risk [22]. A recent meta-study found that a 1-standard deviation increase in cIMT measured in the common carotid artery was associated with CVD events (HR = 1.28, 95% CI = 1.19–1.37) [23]. Several studies demonstrated that the younger the patients or the thicker the cIMT, the stronger the correlation between cIMT and CV risk [23,24]. A recent small prospective study showed that cIMT and CV risk were related in Asians [25]. However, studies conducted in patients who had negative findings on treadmill tests are scarce, and some of the studies that included such patients did not have a sufficient sample size [8,26]. This study supplements these insufficient data. Therefore, patients with increased cIMT should be managed carefully even when they have negative TSE results.

Regarding the relationship between CAC scores and CVD risk, CAC scoring is recommended when risk estimation for CV diseases is uncertain [14]. A recent meta-analysis demonstrated that patients with CAC had increased all-cause mortality (pooled relative risk [RR] 2.13, 95% CI 1.57–2.90, *p* = 0.004) and CV events (pooled RR 2.91, 95% CI 2.26–3.93, *p* < 0.001) compared to patients without CAC [27]. The addition of the CAC score to the transitional risk factor significantly improved CVD prediction in middle-aged and older patients [28]. Risk classification using the CAC score helps with clinical management, especially in patients with a low clinical likelihood of CAD. [29]. However, previous studies have demonstrated the utility of the CAC score in asymptomatic patients [16]. The value of the CAC score in symptomatic patients is less known [22,23,24,25]. In a recent study conducted on patients with stable chest pain, those without CAC had a lower CV risk (HR = 0.08, 95% CI = 0.02–0.30, *p* < 0.001), but the treadmill test was not performed [30]. Combining the treadmill test and CAC scores in low-risk patients, with or without symptoms, helps predict long-term clinical outcomes [31]. Another study suggested that the CAC score is helpful for CVD prediction in symptomatic patients with treadmill-equivocal results [14]. However, the value of the CAC score for symptomatic patients with negative TSE findings has not been studied. This study suggests that an increased CAC score is associated with increased CV outcomes and can be a predictive marker for CV outcomes in symptomatic patients with negative TSE results.

This study is distinctive in that it was conducted on patients with negative TSE but with angina symptoms. Compared to previous studies, the significance of this study is as follows: First, in TSE-negative patients with angina symptoms, atherosclerosis is related to CV outcomes, and atherosclerosis can be assessed by cIMT or CAC scores. Second, an increase in cIMT or CAC scores can be an independent risk factor for CV outcomes in symptomatic patients with negative TSE findings.

### Limitation

There are several limitations in this study. The first limitation is the small sample size. In particular, the number of patients undergoing CAC scoring was small due to various factors, including cost, use of contrast medium, and domestic insurance coverage. Moreover, CAC scoring was conducted only in patients with negative TSE findings; therefore, the CAC score was extremely skewed, resulting in a wide CI for HR. Nevertheless, we demonstrated the value of the CAC score, and its clinical implication was considerable.

Second, the study was conducted at a single center, which limits the generalizability. Follow-up studies using diverse and large samples are needed.

Third, the follow-up median time is only 5.8 years. This is because many patients recently participated in the study. Short follow-up time may cause errors in predicting prognosis. Future studies, including continuous follow-up, are necessary.

## 5. Conclusions

In this study, the increased cIMT and CAC scores were associated with increased CV events in symptomatic patients with negative TSE findings. The cIMT and CAC scores are markers for evaluating atherosclerosis, and patients with atherosclerosis are likely to have a higher CV risk than patients without atherosclerosis. However, because the increase in cIMT or CAC scores indicates the early stages of atherosclerosis, it was controversial whether these abnormalities affect CV outcomes. By selecting symptomatic patients with negative TSE, this study highlights that the presence or absence of atherosclerosis may be a useful indicator for predicting CV events in symptomatic patients without prominent ischemia. This study demonstrated that the increased cIMT and CAC scores in symptomatic patients with negative TSE may be a risk factor for CV events. If additional evidence is available, active lipid-lowering therapy and antihypertensive therapy may be considered for these patients in the future. In conclusion, although TSE-negative, symptomatic patients with increased cIMT or CAC scores should be carefully managed and closely monitored. Prospective studies with various and larger sample sizes will be needed in the future to establish radiological atherosclerosis as an independent risk factor for CVD.

## Figures and Tables

**Table 1:** Baseline characteristics.

	cIMT Group			CAC Scoring Group		
	Normal (n = 1017)	Increased (n = 35)	*p*-Value	Normal (n = 260)	Increased (n = 56)	*p*-Value
Clinical Characteristics						
Age, years	56.0 ± 9.7	62.5 ± 8.6	<0.001	55.7 ± 9.8	62.1 ± 10.1	<0.001
Male, n (%)	544 (53.5)	20 (57.1)	0.670	138 (53.1)	34 (60.7)	0.298
BMI, kg/m^2^	24.9 ± 3.2	26.2 ± 3.3	0.019	24.8 ± 3.1	25.3 ± 3.6	0.243
Smoking, n (%)	199 (19.7)	7 (20.0)	0.965	46 (17.8)	10 (18.2)	0.951
Hypertension, n (%)	402 (39.5)	21 (60.0)	0.015	90 (34.6)	32 (57.1)	0.002
Diabetes, n (%)	213 (20.9)	15 (42.9)	0.002	27 (10.4)	10 (17.9)	0.115
Dyslipidemia, n (%)	506 (49.8)	35 (77.1)	0.001	129 (49.6)	41 (73.2)	0.001
Medications, n (%)						
Antiplatelet agents	202 (19.9)	14 (40.0)	0.004	58 (22.3)	26 (46.4)	<0.001
ACEI/ARBs	202 (19.9)	15 (42.9)	0.001	38 (14.6)	19 (33.9)	0.001
BBs	93 (9.1)	4 (11.4)	0.646	29 (11.2)	5 (8.9)	0.626
Statins	337 (33.1)	19 (54.3)	0.009	80 (30.8)	30 (53.6)	0.001
TSE data						
SBP, mmHg	115.6 ± 14.9	124.5 ± 19.6	0.012	115.0 ± 15.0	120.0 ± 16.9	0.028
DBP, mmHg	67.7 ± 10.6	71.1 ± 14.9	0.192	67.1 ± 11.3	69.6 ± 14.7	0.158
HR, mmHg	70.8 ± 11.4	67.0 ± 8.4	0.014	70.2 ± 11.8	68.7 ± 9.1	0.289
Target HR, %	98.5 ± 9.8	95.9 ± 12.4	0.126	98.5 ± 8.8	95.3 ± 12.4	0.075
METs	11.5 ± 2.1	10.4 ± 2.3	0.004	11.6 ± 2.2	11.0 ± 2.5	0.084
Angina during test, n (%)	514 (52.3)	16 (47.1)	0.544	124 (48.1)	20 (37.7)	0.170

cIMT, carotid intima-media thickness; CAC, coronary artery calcium; BMI, body mass index; ACEI/ARB, renin-angiotensin-converting enzyme inhibitor/angiotensin receptor blocker; BB, beta blocker; TSE, treadmill stress echocardiography; SBP, systolic blood pressure; DBP, diastolic blood pressure; HR, heart rate; MET, metabolic equivalent of tasks.

**Table 2:** Atherosclerotic markers and cardiovascular outcomes.

	cIMT Group			CAC Scoring Group		
	Normal (n = 1017)	Increased (n = 35)	*p*-Value	Normal (n = 260)	Increased (n = 56)	*p*-Value
Atherosclerotic Markers						
Mean cIMT, mm	0.62 ± 0.11	0.98 ± 0.07	<0.001	–		
CAC score	–			0 [0; 5.2]	180.8 [118.15; 410.1]	<0.001
CV outcomes, n (%)	45 (4.4)	7 (20.0)	<0.001	1 (0.4)	14 (25.0)	<0.001
ACS	3 (0.3)	1 (2.9%)	0.016	0	1 (1.8)	0.031
PCI	34 (3.5)	5 (14.7)	0.001	0	13 (23.2)	<0.001
Stroke	7 (0.7)	1 (2.9)	0.151	1 (0.4)	0	0.642
Death	1 (0.1)	0	0.853	0	0	–

cIMT, carotid intima-media thickness; CAC, coronary artery calcium; CV, cardiovascular; ACS, acute coronary syndrome; PCI, percutaneous coronary intervention.

**Table 3:** Univariable and multivariable predictors of the primary cardiovascular outcomes.

	Univariable		Multivariable			
			cIMT		CAC	
	HR (95% CI)	*p*-Value	HR (95% CI)	*p*-Value	HR (95% CI)	*p*-Value
Clinical Characteristics						
Age	1.064 (1.036–1.093)	<0.001	1.068 (1.029–1.108)	<0.001	1.082 (0.997–1.175)	0.060
Male	2.181 (1.282–3.711)	0.004	2.372 (1.250–4.501)	0.008	5.682 (1.127–28.650)	0.035
BMI	1.036 (0.971–1.105)	0.285				
Smoking	1.352 (0.779–2.348)	0.284				
Hypertension	1.921 (1.184–3.115)	0.008	0.837 (0.451–1.550)	0.571	1.800 (0.524–6.187)	0.351
Diabetes	1.832 (1.106–3.034)	0.019	0.749 (0.364–1.542)	0.433	2.726 (0.689–10.785)	0.153
Dyslipidemia	1.085 (0.672–1.752)	0.739				
TSE data						
SBP	1.028 (1.013–1.043)	<0.001	1.023 (1.000–1.046)	0.045	1.040 (1.001–1.081)	0.045
DBP	1.033 (1.012–1.054)	0.002	1.026 (0.996–1.058)	0.093	1.028 (0.981–1.077)	0.253
Heart rate	0.968 (0.947–0.990)	0.005	0.959 (0.930–0.989)	0.009	0.998 (0.931–1.069)	0.946
Target heart rate, %	0.969 (0.947–0.991)	0.007	0.969 (0.943–0.995)	0.020	0.972 (0.909–1.038)	0.396
METs	0.914 (0.824–1.014)	0.089				
Angina during test	1.717 (1.017–2.898)	0.043	1.741 (0.940–3.225)	0.078	1.081 (0.276–4.228)	0.911
Atherosclerotic markers						
Increased cIMT	4.772 (2.151–10.586)	<0.001	2.939 (1.241–6.960)	0.014	–	
Increased CAC	72.917 (9.578–555.097)	<0.001	–		45.192 (5.497–371.505)	<0.001

cIMT, carotid intima-media thickness; CAC, coronary artery calcium; HR, hazard ratio; CI, confidence interval; BMI, body mass index; TSE, treadmill stress echocardiography; SBP, systolic blood pressure; DBP, diastolic blood pressure; METs, metabolic equivalent of tasks.

## Data Availability

The raw data supporting the conclusions of this article will be made available by the authors on request.

## Supplementary Materials

The following supporting information can be downloaded at: https://www.mdpi.com/article/10.3390/biomedicines12092151/s1, Table S1: Coronary angiography in patients with coronary artery disease; Table S2: Primary cardiovascular outcomes for absolute value of atherosclerotic markers; Figure S1: The cutoff value of coronary artery calcium score.
