# Supplementary material for: Assessing Cardiovascular Risk with Coronary Artery Calcium and Carotid Intima-Media Thickness in Patients with Negative Stress Echocardiography
Source: Biomedicines. 2024 Sep 23;12(9):2151. doi: 10.3390/biomedicines12092151 (PMC11429111; doi:10.3390/biomedicines12092151)
Supplement: Supplementary file 1 [file biomedicines-12-02151-s001.zip › biomedicines-3196860-supplementary.pdf]

Table S1. Coronary angiography in patients with coronary artery disease

No.	CAG findings	No.	CAG findings
**Acute coronary syndrome**			
1	**Diagnosis: STEMI**<br>dRCA: near total occlusion<br>mLAD: 50% stenosis<br>dLCX: 80% stenosis<br>→ PCI at dRCA	4	**Diagnosis: STEMI**<br>dRCA: total thrombotic occlusion<br>mLAD: 60% stenosis<br>→ PCI at dRCA
2	**Diagnosis: unstable angina**<br>mdLAD: 75% stenosis<br>→ PCI at mdLAD	5	**Diagnosis: STEMI**<br>mLCX: total thrombotic occlusion<br>→ PCI at mLCX
3	**Diagnosis: unstable angina**<br>pRCA: 95% stenosis<br>→ PCI at pRCA<br>* CAG 2 years ago<br>Borderline disease at mLAD	6	**Diagnosis: NSTEMI**<br>dRCA: 90% stenosis<br>→ PCI at dRCA<br>* CAG 10 years ago<br>PD: 60% stenosis<br>→ failed PCI
**Coronary revascularization**			
**One-vessel disease**			
1	mRCA: CTO, collateral flow from LCX<br>→ PCI at mRCA	13	dLCX: 90% stenosis<br>→ POBA at dLCX
2	dLAD: 80% stenosis<br>→ DEB at dLAD	14	mLAD: 65% stenosis<br>→ PCI at mLAD
3	OM1: 90% stenosis<br>→ PCI at OM1	15	dLCX: 90% stenosis<br>→ PCI at dLCX
4	pLAD: 80% stenosis<br>→ PCI at pLAD	16	mRCA: 75% stenosis<br>→ PCI at mRCA
5	dLCX: 70% stenosis<br>→ PCI at dLCX	17	OM3: 90% stenosis<br>→ PCI at OM3
6	mdLAD: 70% stenosis<br>→ PCI at mdLAD	18	LM to pLAD: 75% stenosis<br>→ PCI at LM to pLAD
7	dLAD: 70% stenosis<br>→ PCI at dLAD	19	dLAD: 70% stenosis<br>→ PCI at dLAD
8	dLCX: 70% stenosis<br>→ PCI at dLCX	20	mLCX: 60% stenosis<br>→ PCI at mLCX
9	mLCX: 70% stenosis<br>→ PCI at mLCX	21	mLAD: 70% stenosis<br>→ PCI at mLAD
10	pRCA: 70% stenosis<br>→ PCI at pRCA	22	pRCA: 60% stenosis<br>→ PCI at pRCA
11	pLAD: 70% stenosis<br>→ PCI at pLAD	23	mLAD: CTO, collateral flow from RCA<br>→ PCI at another hospital
12	mLCX: 90% stenosis<br>→ PCI at mLCX	24	**Initial CAG (2013.6)**<br>Minimal disease<br>**Follow-up CAG (2022.3)**<br>mLAD: 80% stenosis<br>→ PCI at mLAD

**Two-vessel disease**			
25	mRCA: 70% stenosis<br>dLCX: 70% stenosis<br>→ PCI at mRCA	31	mLAD: 55% stenosis<br>mLCX: 70% stenosis<br>→ PCI at mLAD and mLCX
26	dLAD: 60% stenosis<br>OM1: 60% stenosis<br>→ POBA at dLAD and PCI at OM1	32	mdRCA: total occlusion<br>dLAD: 60% stenosis<br>→ PCI at mdRCA and dLAD
27	mLAD: 70% stenosis<br>dLCX: 60% stenosis<br>RI: 90% stenosis<br>→ PCI at mLAD, dLCX and RI	33	mLAD: CTO, bridging collateral flow to dLAD<br>pRCA: 50% stenosis<br>→ PCI at another hospital
28	dLCX: total occlusion<br>mLAD: 75% stenosis<br>→ PCI at dLCX and mLAD	34	dRCA: 90% stenosis<br>mLCX: 70% stenosis<br>→ PCI at dRCA and mLCX
29	mdLCX: 70% stenosis<br>mdRCA: 85% stenosis<br>→ PCI at mdLCX & and mdRCA	35	pRCA: 75% stenosis<br>D2: 90% stenosis<br>→ PCI at pRCA
30	mLAD: 65% stenosis<br>pRCA: 60% stenosis<br>→ PCI at mLAD and pRCA	36	mLAD: 90% stenosis<br>mLCX: 50% stenosis<br>→ PCI at mLAD
**Three-vessel disease**			
37	pRCA: 90% stenosis<br>mLAD 70% stenosis<br>pmLCX: 60% stenosis<br>→ PCI at pRCA and mLAD	38	pRCA: 80% stenosis<br>dLCX: CTO, collateral flow from RCA<br>mLAD: 60% stenosis<br>→ PCI at pRCA
39-50	No data on coronary artery as CAG and revascularization were performed at another hospital		

CAG, coronary angiography; STEMI, ST elevation myocardial infarction; RCA, right coronary artery; LAD, left anterior descending artery; LCX, left circumflex artery; PCI, percutaneous coronary intervention; NSTEMI, non-ST elevation myocardial infarction; d, distal; m, mid; p, proximal; PD, posterior descending artery; POBA, plain old balloon angioplasty; DEB, drug-eluting balloon; OM, obtuse marginal artery; LM, left main coronary artery; CTO, chronic total occlusion; RI, ramus intermedius artery.

Table S2. Primary cardiovascular outcomes for absolute value of atherosclerotic markers

	Univariable		Multivariable			
			cIMT		CAC	
	HR (95% CI)	p-value	HR (95% CI)	p-value	HR (95% CI)	p-value
**Clinical characteristics**						
Age	1.064 (1.036–1.093)	<0.001	1.058 (1.018–1.099)	0.004	1.106 (1.020–1.199)	0.015
Male	2.181 (1.282–3.711)	0.004	2.244 (1.182–4.260)	0.013	3.511 (1.016–12.136)	0.047
BMI	1.036 (0.971–1.105)	0.285				
Smoking	1.352 (0.779–2.348)	0.284				
Hypertension	1.921 (1.184–3.115)	0.008	0.787 (0.430–1.442)	0.438	1.120 (0.337–3.722)	0.853
Diabetes	1.832 (1.106–3.034)	0.019	0.747 (0.366–1.522)	0.422	1.718 (0.384–7.681)	0.479
Dyslipidemia	1.085 (0.672–1.752)	0.739				
**TSE data**						
SBP	1.028 (1.013–1.043)	<0.001	1.022 (1.000–1.046)	0.053	1.051 (1.011–1.094)	0.045
DBP	1.033 (1.012–1.054)	0.002	1.026 (0.995–1.058)	0.098	1.031 (0.974–1.090)	0.253
Heart rate	0.968 (0.947–0.990)	0.005	0.958 (0.928–0.989)	0.008	0.973 (0.916–1.033)	0.946
Target heart rate, %	0.969 (0.947–0.991)	0.007	0.969 (0.943–0.996)	0.024	0.963 (0.910–1.019)	0.396
METs	0.914 (0.824–1.014)	0.089				
Angina during test	1.717 (1.017–2.898)	0.043	1.702 (0.925–3.132)	0.087	0.826 (0.226–3.013)	0.911
**Atherosclerotic markers**						
cIMT	92.987 (16.576-521.630)	<0.001	17.713 (2.180-143.953)	0.007	–	
CAC	1.002 (1.001-1.002)	<0.001	–		1.002 (1.001-1.003)	0.001

cIMT, carotid intima-media thickness; CAC, coronary artery calcium; HR, hazard ratio; CI, con-fidence interval; BMI, body mass index; TSE, treadmill stress echocardiography; SBP, systolic blood pressure; DBP, diastolic blood pressure; METs, metabolic equivalent of tasks.

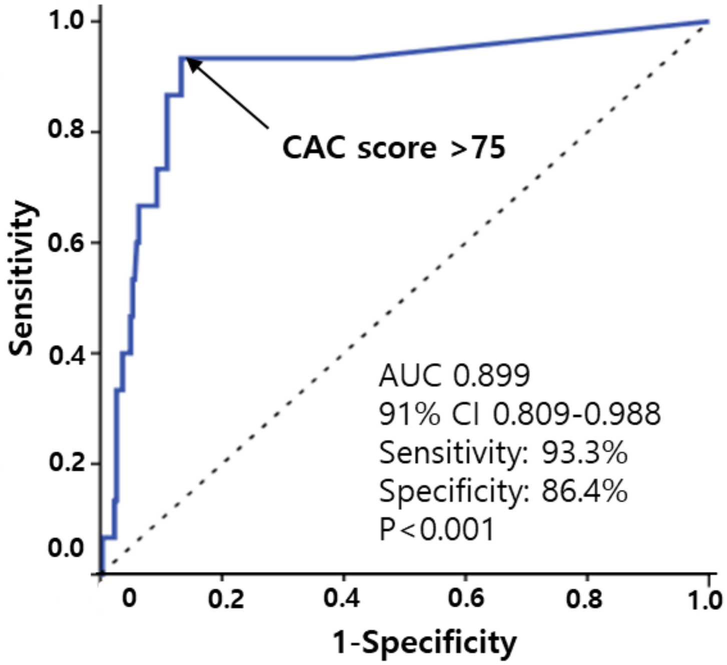

Figure S1. The cutoff value of coronary artery calcium score. CAC, coronary artery calcium; AUC, area under curve; CI, confidence interval.
